# Viruses and neurodegeneration

**DOI:** 10.1186/1743-422X-10-172

**Published:** 2013-05-31

**Authors:** Li Zhou, Monica Miranda-Saksena, Nitin K Saksena

**Affiliations:** 1 Retroviral Genetics Division, Center for Virus Research, Westmead Millennium Institute, Westmead Hospital, The University of Sydney, Westmead NSW 2145, Sydney Australia; 2 HSV Transport Laboratory, Westmead Millennium Institute, Westmead Hospital, Westmead NSW 2145, Sydney, Australia

**Keywords:** Neurodegenerative diseases, Virus, Alzheimer’s disease, Parkinson’s disease, Multiple sclerosis, Amyotrophic lateral sclerosis

## Abstract

Neurodegenerative diseases (NDs) are chronic degenerative diseases of the central nervous system (CNS), which affect 37 million people worldwide. As the lifespan increases, the NDs are the fourth leading cause of death in the developed countries and becoming increasingly prevalent in developing countries. Despite considerable research, the underlying mechanisms remain poorly understood. Although the large majority of studies do not show support for the involvement of pathogenic aetiology in classical NDs, a number of emerging studies show support for possible association of viruses with classical neurodegenerative diseases in humans. Space does not permit for extensive details to be discussed here on non-viral-induced neurodegenerative diseases in humans, as they are well described in literature.

Viruses induce alterations and degenerations of neurons both directly and indirectly. Their ability to attack the host immune system, regions of nervous tissue implies that they can interfere with the same pathways involved in classical NDs in humans. Supporting this, many similarities between classical NDs and virus-mediated neurodegeneration (non-classical) have been shown at the anatomic, sub-cellular, genomic and proteomic levels suggesting that viruses can explain neurodegenerative disorders mechanistically. The main objective of this review is to provide readers a detailed snapshot of similarities viral and non-viral neurodegenerative diseases share, so that mechanistic pathways of neurodegeneration in human NDs can be clearly understood. Viruses can guide us to unveil these pathways in human NDs. This will further stimulate the birth of new concepts in the biological research, which is needed for gaining deeper insights into the treatment of human NDs and delineate mechanisms underlying neurodegeneration.

## 

Neurodegenerative diseases are chronic degenerative diseases of the central nervous system (CNS) resulting from neurodegenerative processes. They are characterized by chronic progressive loss of structure and function of neurons. They are increasingly common disorders, which strike in mid-to-late adult life. They affect 37 million people worldwide and are the fourth leading cause of death in the developed countries and are becoming increasingly significant in the developing countries as well. The incidence rate is expected to become higher as the lifespan increases. Despite intensive research, the underlying causes of most neurodegenerative diseases remain poorly understood. Several intracellular mechanisms are involved in classical neurodegenerative diseases: protein degradation, mitochondrial dysfunction, defective axonal transport and apoptosis. The pathological mechanisms underlying neurodegenerative disorders in humans have gained renewed attention, not only because human NDs are growing cause of concern and disability in ageing humans globally in ageing humans, but also in part due to the evolving nature of NDs in humans, possible pathogen involvement and recent evidence from proteomic and genomic fronts showing similarities between virally-induced NDs and classical NDs in humans [1-3].

Immune activation within the central nervous system (CNS) is classically observed in several immune-mediated disorders, viral infections and also in human neurodegenerative diseases. Although, often the immune responses may contribute directly or indirectly to neuronal damage, not all immune responses in the CNS are damaging because they assist in repair and regeneration. But heightened immune responses can be indicative of underlying pathogenic process, which may shift the delicate balance between beneficial and detrimental response [2,3].

As global research on neurodegenerative diseases progresses on various fronts, many similarities between classical neurodegenerative diseases and virus-mediated neurodegeneration are becoming apparent at both anatomic and sub-cellular level [1,4,5]. Supporting these observations, a number of viruses have been shown to be associated with different types of neurodegenerative diseases, which can guide us underpin mechanisms in neurodegeneration (Table 1). We know that neurotropic viruses are able to induce considerable neuronal dysfunction by inducing alterations and degenerations of neurons both directly and indirectly leading to devastating effects (Table 1). Viruses can injure neurons by direct killing, cell lysis and by inducing apoptosis. Thus regardless of the route of entry of neurotropic viruses into the CNS, viral infection of the CNS leads to activation of both innate and adaptive immune responses. Viral antigens preferentially activate TLRs 3, 7 and 8 are driving innate and adaptive immune responses and leading to neuronal damage, which occurs through direct damage, killing, release of free radicals, cellular activation and inflammation [2]. In contrast, the immunocompetent host is able to clear viruses rapidly.

**Table 1:** Association of viruses and neurodegeneration

**Virus**		**Family**	**Species**		**Reported neurological manifestations**	**Reference**
DNA		Herpesviridae	Herpes simplex virus (HSV)		Cognitive changes	[6]
					Neuronal destruction	
			Human herpesvirus 6 (HHV6)		Meningoencephalitis and leucoencephalitis	[7]
					Dead and dying neurons undergoing neuronophagia	
			Epstein-Barr virus (EBV)		Grey-matter atrophy	[8]
					Encephalopathy and acute quadriparesis Anterior horn cell degeneration	
			Cytomegalovirus (CMV)		Transverse myelitis	[9]
			Variecella zoster virus (VZV)		Infection of trigeminal ganglion	[10]
		Polyomaviridae	JC virus		Infection of oliodendrocytes, astrocytes and neurons	[11]
RNA	(−)ssRNA Virus	Bornaviridae	Borna disease virus		Patent infection of the limbic system	[12]
		Orthomyxoviridae	Influenza virus	H3N2	Amyotrophy, MS flares, encephalitis, encephalopathy, myelopathy, GBS, seizures, relapsing delirium,	[13,14]
				H1N1	Delirium, cycloplegia, encephalitis lethargica, GBS, encephalopathy, seizures, ataxia	[15-19]
				H2N2	Encephalitis, seizures, muscle paralysis, GBS	[20,21]
				H5N1	Viral neurotropism in animals	[18]
		Paramyxoviridae	Measles		Myelin damage	[22]
		Rhabdoviridae	Rabies virus		Cognitive changes, neuronal destruction	[6]
	(+)ssRNA	Flaviviridae	West Nile Virus		Encephalitis	[23]
			Japanese encephalities B virus		Neuronal death	[24]
			St. Louis virus		Juvenile parkinson disease, encephalitis	[25,26]
	dsRNA	Picornaviridae	Poliovirus		Encephalitis	[27]
			Echo virus		Meningitis, neuro-muscular diseases	[28]
			Coxsackie virus		Encephalitis	[29]
			Enterovirus 71		Encephalitis, aseptic meningitis, brain stem enceptitis and motor neuron death	[30]
	(+)ssRNA	Retroviridae	HIV		HIV associated dementia	[31]

Even though several biological studies, with numerous examples, implicate variety of viruses in classical NDs, the direct role of viruses in human NDs remains partially proven and controversial at best. Because of this controversy surrounding the role of several viruses in classical human NDs, the primary objective of this review is to provide a detailed and a unique snapshot of how viruses lead to neurodegeneration in humans and their possible involvement in classical human NDs, which will shed light not only on a profound understanding of viruses as mediators or modulators of neurodegenerative diseases, but also on the development of future strategies to treat them.

## Viral association in human NDs

### a. Herpesviridae and their association with Alzheimer’s disease (AD) (herpes simplex virus-1(HSV-1 type HHV-1)

HSV-1 is one of the most common viruses in the general population and can cause latent infection in neurons for life-long. It belongs to the family Herpesviridae, which is a large family of double-stranded DNA viruses [32,33]. HSV-1 is neurotropic and neuro-invasive with high neuro-virulence and grows well *in vitro* in both neuronal cell lines and dorsal root ganglia. Primary sympathetic neuronal cultures support HSV-1 infections, which resemble natural latency. *In vivo*, HSV-1 establishes life-long latency in peripheral neurons where productive replication is suppressed. While periodic reactivation results in productive infection, the molecular, genomic and proteomic basis of neuronal latency remains poorly understood.

AD is the most common form of dementia. There are 26.6 million patients suffering AD worldwide and according to the World Health Organization, the prevalence would increase to 0.441% by 2015 and to 0.556% by 2030 (WHO). Because it can’t be cured, it is one of the most expensive diseases to manage and treat in the developed countries [34]. AD is characterized by loss of neurons and synapses in the cerebral cortex and certain subcortical regions. One of the most notable pathological features is the appearance of plaques and tangles of neurofibrils within brain nerves. AD has been identified as a protein mis-folding disease as both plaques and tangles involve the deposition and accumulation of abnormally folded A-beta and tau proteins [35,36]. Precisely how the disturbances of production and aggregation of the beta-amyloid peptide leads to the pathology of AD is poorly understood. The amyloid hypothesis suggests the accumulation of beta-amyloid peptides as the central event triggering neuron degeneration. Accumulation of aggregated amyloid fibrils, which are believed to be the toxic form of the protein responsible for disrupting the cell’s calcium ion homeostasis, induces programmed cell death (apoptosis) [37]. It is also known that βamyloid selectively builds up in the mitochondria in the cells of Alzheimer’s-affected brains, and it also inhibits certain enzyme functions and the utilization of glucose by neurons[38]. In addition, various inflammatory processes and cytokines possibly play significant role in the pathology of Alzheimer’s disease. Inflammation is indicative of tissue damage in any disease, but it is likely that it may be a marker of an immunological response [39]. Yet, the precise cause of AD is not well understood and recently brain infections have been suggested as a possible trigger or cofactor(s) in AD. Several viruses have been linked to AD (Table 1), with HSV-1 being the most popular and well studied in relation to AD.

The presence of HSV-1 DNA in elder brain, especially for APOE gene carriers, is a risk factor for AD [40]. Its seropositivity reactivation has also been shown to significantly correlate with incident AD [41]. Furthermore, HSV-1 infection in AD brain has been detected by electron microscopy and immunohistochemistry in a rapid progressor with AD, indicating the significant role of reactivation of HSV in AD progression [42]. In addition, Wozniak et al., has further demonstrated the role of HSV-1 in AD by localizing HSV-1 DNA in amyloid plaques and detected higher association between viral DNA and Amyloid beta plaques in AD patients’ brains compared to normal ageing brains (72% versus 24%, p<0.001), suggesting HSV-1 to be a possible major cause of amyloid plaques and a possible etiological factor in AD [43]. Moreover, short contiguous amino acid stretches in proteins expressed by HSV are homologous to APOE4, clusterin, and many other gene products highly relevant to AD, also suggesting the role of HSV as a causative agent that is involved in AD development [44].

The possible mechanisms by which HSV-1 triggers AD have been studied in some detail. It has been found that isolated intracellular HSV1 particles physically associated with amyloid precursor protein (APP), through which the viruses exploit to travel to the cell surface [45,46]. APP is an integral membrane protein highly expressed in the synapses of neurons and best known as the precursor molecule whose proteolysis generates beta amyloid (Aβ). Abnormal deposition and accumulation of Aβ and tau proteins serves as the primary cause of plaques and tangles formation, which is one of the most notable pathological features in the AD brain [35,36]. Cheng et al., has further proven that HSV1 is specifically co-localized with APP to facilitate viral transport and also alters normal APP transport, distribution, and even microtubule networks and their stability [47]. HSV-1 infection has also been shown to promote neurotoxic Aβ accumulation [48-50], tau phosphorylation [51] and cleavage [52]*in vitro*. Apart from direct interaction, HSV1 infection has also been found to interfere with post-transcriptional regulation by up regulating microRNA-146a, which is significantly involved in AD [53].

With the compelling evidence of HSV-1 association with AD, HSV-1 infection in people over 50 years of age is definitely a risk factor or co-factor at least in AD. Therefore, it remains to be seen if the administration of antiviral agents to patients with mild AD has any direct effect on improvement and general well being of patients with AD. This may provide a further confirmation of HSV-1 involvement in AD.

### b. Multiple sclerosis (MS) and its relationship with the roseolovirus (type HHV-6), eppstein Barr virus (EBV type HHV-4) and varicella zoster virus (VZV type HHV3)

#### Roseolovirus (HHV-6)

MS, commonly known as a chronic inflammatory demyelinating disease of the CNS, is the most common cause of neurological disability in young adults. It recently has been classified as neurodegenerative diseases due to: 1. The axonal loss and dysfunction occuring at very early stages of MS [54]; 2. The clinical disability of MS correlates better with the extent of total axonal damage, loss and brain atrophy [55-57]; and 3. Critical loss of axon density during disease progression [58,59]. It has four clinical subtypes, among which relapsing-remitting subtype describes the initial course of 80% of individuals with MS [60]. Although the mechanisms in MS disease process are well known, the underlying causes remain poorly understood. Unlike other neurodegenerative diseases, MS might be a consequence of a combination of genetic, environmental and possibly infectious factors, but so far no plausible explanation has proven definitive. In this context it is important to mention that viruses have long been suggested to be involved in the etiology of MS [61].

Among the most well described viruses, growing body of evidence suggests that HHV-6 can play a direct and an indirect role in MS either as an activator of viruses such as EBV and human endogenous retrovirus-W (HERV-W). Noteworthy is its ability to infect almost 100% of the general population worldwide [62] and remain latent in approximately 90% of the adults [63]. Although it remains rare in causing primary infections in adults, and the re-activation is often asymptomatic in the immuno-competent setting, it can trigger serious complications in immunosuppressed patients [64]. HHV6 protein is reported to be expressed in oligodendrocytes in active MS plaques [65]. Recently, Alvarez-Lafuente et al., also showed HHV-6 DNA in the spinal fluid of a subset of MS patients more frequently than in those with other neurological disease [66]. In support of these findings, it has been shown that HHV-6 serum DNA levels diminish with interferon-β treatment and patients who do not clear HHV-6 infection during interferon therapy have a poor prognosis [67]. The virus is known to periodically re-activate from its latent state and Chapenko et al., have shown a significant association between HHV-6 infection and MS in addition to a correlation between HHV-6 reactivation and disease activity in relapsing/remitting and secondary progressive MS [68]. In addition, HHV-6 presents more frequently in blood and serum during relapses compared to during remission and this, in turn, correlates with higher risk of severe relapse and poor response to interferon-β therapy [67]. Further, increased IgM serum antibody responses to HHV-6 early antigen (p41/38) in patients with relapsing-remitting MS (RRMS), compared to patients with chronic progressive MS (CPMS), other neurologic diseases (OND and autoimmune diseases (OID), and normal controls have been demonstrated. Interestingly these antibody studies were further supported by the detection of HHV-6 DNA from samples of MS serum indicating active viral infection [69]. Furthermore, the validity of connection between HHV6 and MS has also been replicated in animal models. Investigators from the UCSF and the NINDS induced brain lesions and MS-type ambulatory deficits in marmosets after exposing them to intranasal injections of HHV-6A virus [70,71].

Recently, Vandenbroeck et al., [72] analyzed IRF5 polymorphisms rs4728142 and rs3807306 in MS predisposition, by combining the five European datasets, which include three Spanish replication cohorts. Combining these datasets have shown that the T allele of rs3807306 is a significant marker for both susceptibility to MS and infection with HHV-6 and also constitutes a promising marker for response to IFN-β therapy. This study also suggests complex interactions centering on the IRF5 gene, which, upon further investigation may help in understanding the interplay between genes and virus in predisposition to MS [72]. In this context, it is important to recall that HHV-6 has been reported to be associated with MHC class II transactivator (MHC2TA) rs4774C (MHC2TA rs4774C and HHV-6A)[73]. Further stressing the role of virus in MS in manipulating the host immune system is the evidence for lymphoproliferative responses to HHV-6A together with the evolution of lesions in MRI in relation to HHV-6 infection has been found in MS patients [74], which appears to be greater in MS patients (67%) than in controls (32%). Such observations are critical in tracing the possible links of a pathogenic etiology in MS.

#### EBV (HHV-4)

Epstein-Barr virus (EBV), another neurotropic virus in the HHV family, has been first linked to MS in 1979 by Fraser and colleagues, demonstrating increased tendency of peripheral blood lymphocytes from active MS patients toward spontaneous *in vitro* EBV-induced B lymphocyte transformation [75]. Over 90% of all populations get infected within the first decades of life with EBV, which can latently persist in B-lymphocytes for the lifetime of the human host. The higher risk has been reported when EBV seroconversion occurs at later stage in young adults compared to first decades [76]. Contrary to these findings, a recent longitudinal study, comparing 305 individuals who developed MS and 610 healthy adults, has shown no cases of MS before EBV infection and similar rate of MS after seroconversion compared to who were already EBV positive [77]. These results have clearly shown the temporal relationship between MS risk and primary EBV infection, although other alternative interpretations for this exist well. In addition, a meta-analysis of case-controlled observational studies published in January 2009 has demonstrated the statistically significant association between MS and an exposure to EBV by determining the anti-VCA, anti-EBNA IgG, anti-EBNA-1 IgG antibodies [78]. The strong association between MS and anti-EBNA IgG, anti-EBNA-1 IgG antibodies was also supported by recent large prospective study, which included 222 individuals with MS and 444 healthy young adults) [79]. However, due to the defined subcellular localization of EBV genes, EBV DNA is hardly detected in internal compartment including CSF and plasma while it can easily be detected in saliva [80-83]. Therefore, it is not surprising that some of the studies failed to identify the EBV DNA or RNA in CSF and CSF plasma cells [84,85], but the increased titer of EBV antibody is good evidence of link between EBV recent infection and MS relapse. The relationship between EBV and MS has been further supported by the presence of EBV virus-encoded small RNA in MS brains [86].

Although compelling evidence linking EBV infection to MS risk has been described in literature, the underlying mechanisms still remain unknown. Several hypotheses that could explain the potential contribution of EBV to the pathogenesis of MS. Firstly, the EBV specific CD8+ T cell responses initiate and sustain tissue damage. This idea is supported by Jilek et al. [87], who showed increase in EBV- specific CD8+ T cells response at the early stages of MS patients when compared to healthy adults. But Pender et al., have reported conflicting results [88] and Gronen et al., failed to locate any differences at all [89]. So far, according to the author’s knowledge, there is no study identifying EBV within the CNS of MS patients. Secondly, EBV triggers autoimmunity in MS by molecular mimicry between EBV and myelin antigens [90,91] or infecting auto-reactive B cells [92]. Furthermore, EBV infection can facilitate brain infiltration by activating macrophages and lymphocytes and inducing cytokines [93]. And, lastly EBV can mediate transactivation of the expression of HERV elements [94]. Therefore, further experiments are needed to elucidate the mechanisms how EBV is involved in MS occurrence and its progression.

#### Varicella zoster virus (VZV) (HHV3)

VZV shares considerable genome homology with HSV. As discussed in the subsequent sections for H1N1 Influenza A viruses, the epidemiology of MS resembles that of VZV attack [95]. In addition, the antecedent of VZV appears to be more frequent in MS patients compared to controls [96,97]. Sotelo et al., have demonstrated more abundant presence of VZV DNA and VZV-like viral particles (visualized by electron microscopy and precipitated with anti-VZV antibodies) in the cerebrospinal fluid (CSF) of MS patients during relapse compared to during remission and the control subjects, suggesting the possible involvement of VZV in the pathogenesis of MS [98,99]. Recently, they have further shown the amount of DNA and the number of viral particles in the CSF of progressive MS patients fell in between relapse and remission, indicating VZV not only plays a possible role in MS onset but in its progression as well [100]. Supporting this, the amount of viral DNA during relapse was hundreds of times higher in the CSF than in peripheral blood mononuclear cells (PBMC). More interestingly, viral DNA as well as viral particles in the CSF and PBMC decreased dramatically after acute relapse [98]. However, intriguingly, VZV DNA is not present in the CSF of MS patients with initial demyelinating event [101], indicating that VZV infection might take place at some stage of MS rather than before it, and then plays an accelerating role in MS relapse? Or even after the occurrence of MS, patient is more susceptible to VZV. Further experiments at different stages of MS on a bigger scale are needed to elucidate the nexus between MS and VZV infection.

Although there are other viruses reported to be involved in MS, the astounding evidence of HHV participation in MS strongly implies either herpes virus structure, or their lytic reactivation ability, or their immune evasion capability playing an important role in MS. In addition, a combination of host genetic factors might also contribute to susceptibility to individual viruses belonging to this family. This hypothesis can be only viable when longitudinal experiments with more specific aims are carried out.

### c. H1N1 Influenza a viruses and their possible association with Parkinson’s disease (PD)

PD is the second most common age-related neurodegenerative disorder after AD and it influences more than 1 million people in the US and prevalence is predicted to triple over the next 50 years due to increase in lifespan. Its primary characteristic is the loss of dopamine-secreting cells in the *pars compacta* region of the *substantia nigra*[102]. The cause remains unknown for majority of PD cases although small percentage of diagnosed PD has a genetic causation. Apart for several possible biological, environmental and genetic causes linked to PD, it has also been linked to influenza virus first time after the occurrence of encephalitis lethargica, an entity displaying Parkinson’s disease (PD) signs and symptoms. Influenza A viruses are negative sense, single-stranded, segmented RNA viruses, belonging to the Orthomyxoviridae family. By yearly epidemic, it can cause up to 500,000 deaths worldwide [103]. So far, it has caused five flu pandemics. 1918 flu pandemic, caused by H1N1 subtype, is the most lethal one, which killed 20–100 million people.

Increased incidence of PD in relation to the 1918 H1N1 influenza A pandemic has been well documented [104-106],including the incidence in years around 1918 and also the incidence of people who were born around 1918 [105,107,108]. Followed by the 1918 Spanish influenza pandemic, the association of PD with influenza A has become more evident, yet not completely proven [109]. It has been shown by Jang *et al.*[110] that certain viruses can enter the CNS and are capable of directly inducing pathological alterations reminiscent of those observed in Parkinson’s disease. These include, loss of dopaminergic neurons in the SNpc, long-lasting activation of microglia in areas infected by virus, as well as upregulation of alpha-synuclein (with aggregation) in these same regions. In addition, the possible involvement of H1N1 as an etiological factor in the development of Parkinson’s disease has been offered as an alternative hypothesis due to the presence of Parkinsonian clusters, where small groups of people in close contact with each other develop Parkinson’s disease in significantly higher incidences that would be predicted from the general population, which affirms this particular influenza virus could be the etiological cause of the Parkinsonian pathologies [110]. At least with the virus Jang *et al.* studied, the pattern of infection mimicked the hierarchical progression of the disease as described by Braak, with infection starting in the enteric nervous system, traveling into the CNS via the vagus nerve where it first infects that solitary and dorsal motor nucleus of X, and then spreading from the brain stem into higher regions of the CNS.

So far, several influenza A viruses have been shown to be neurotropic [111-116], including H1N1 and H5N1. In addition, *substantia nigra*- the most affected brain region in PD patients, has been shown to be targeted by A/WSN/33 (H1N1) before its spread in the CNS [117]. Moreover, clear motor deficits have been shown in poultry [118] as well as humans [119,120] following H5N1 infection. Using the C57BL/6J mouse, Jang et al., has found A/Vietnam/1203/04 H5N1 virus can enter CNS from peripheral nervous system and induce Parkinsonian symptoms (e.g. loss of dopaminergic neurons) and microglia activation as well as alpha-synuclein phosphorylation and aggregation [110]. Recently, Rohn *et al.*[121] have also demonstrated the presence of influenza A virus within the *substantia nigra pars compacta* (SNpc) from postmortem PD brain sections. They also identified co-localized influenza A and immune cells with caspase-cleaved Beclin-1 within the SNpc, which clearly indicated the role of neuro-inflammation with influenza A virus’s involvement in PD pathogenesis. However, no direct correlation between virus and PD pathology degree and clinical bragging has been made, so it is hard to conclude how much this virus contributes to the disease onset and its progression.

### d. HIV and its role in HIV associated neurocognitive disorders (HAND)

HAND are common neurological disorders associated with HIV infection and AIDS. According to current diagnostic criteria, there is asymptomatic neurocognitive impairment (ANI), HIV-associated mild neurocognitive disorder (MND), and HIV-associated dementia (HAD). Among them, HAD is the most severe form of HAND in terms of its functional impact. Its neuropathologic features are perivascular macrophage infiltration, multinucleated giant cells, activated microglias/microglia nodules, pronounced reactive astrocytosis, myelin pallor on microscopic sections and neuron loss. The pathogenesis of HAD is not certain yet, but the neurotoxicity of viral proteins, mononuclear phagocytes activation, cytokine/chemokine and other soluble factors all show involvement [122,123].

Although with the advent of highly active antiretroviral therapy (HAART), the incidence has considerably dropped initially, the prevalence of HIV-associated cognitive impairment appears to be on the rise due to the increased life span of HIV+ population. So far, it is still the most devastating viral-associated neurodegenerative disease because it impacts more than 30% of HIV-infected population. Compelling evidence has been shown that productive HIV infection in the brain is essential for the occurrence and deterioration of HAD [124]. Recently, it has been shown that HIV-1 can replicate in at least macrophage and CCR5+ T cells within the CNS in relation to the development and progression of dementia [125]. Further, the evidence has also demonstrated that HIV gp120 can induce neuronal apoptosis by enhancing IA via CXCR4-PKC signaling [126] and HIV Tat protein can cause neuronal dysfunction by disrupting miRNAs expression [127].

One of the most striking features of HAD is that it shares the common anatomical substrate (hippocampus and *substantia nigra*) with AD and PD. In addition, it also shares dopamine deficiency and testosterone deficiency with PD. Also, there is commonality between HAD and AD in many aspects, such as insulin resistance, raised cholesterol especially in mid-life, and testosterone deficiency, the cytokines that are highly secreted, elevations in lipopolysaccharide concentrations, APOE4 [128]. Thus, the manifestation of AD and PD in ageing HIV patients is not surprising.

It has also been reported that some HAD patients have MS and ALS-like syndromes [129,130]. Our studies further supported the similarity between HAD and classical neurodegeneration on proteomic, genomic as well as transcriptional regulation levels. We have studied the brain proteome of 9 HIV-associated dementia and 5 HIV non-dementia patients found that 31 proteins to be significantly altered in certain areas of the brain [1]. Worthy to note, over 90% of the 31 proteins characterized from the HIV-infected brains with HAD have already been identified previously in studies involving other neurological diseases such as AD, PD, schizophrenia, Lewy body dementia, etc. (Table 2). This proteomic overlap between proteins found in HAD brains and classical NDs is not only striking, but its also shows how mechanistically similar are the pathways in viral and non-viral neurodegenerative diseases. The take home lesson is that further research in this area, using viruses as models, can guide us in unraveling mechanisms underlying neurodegenerative diseases, which is vital to understand if new strategies for treatment are to be developed. In addition, they can also determine any role pathogenic etiologies play in classical NDs in humans.

**Table 2:** Intersecting pathways and proteins between HIV dementia and classical neurodegenerative diseases in humans

**KEGG pathway**	**Spot no**	**Ac. No.**	**Name**	**Ratio**	**P value**	**Related neurological disease**
Glycolysis/ Gluconeogenesis pathway	387	P09972	Fructose-bisphosphate aldolase C [Homo sapiens]	1.69	0.0009	Schizophrenia, bipolar disorder, and depression [131]
	471	P00338	L-lactate dehydrogenase A chain [Homo sapiens]	1.5	0.039	
	439	P07195	L-lactate dehydrogenase B chain (LDH) [Homo sapiens]	1.43	0.028	
	412	P14550	Alcohol dehydrogenase [NADP+][Homo sapiens]	1.36	0.028	
	605	P60174	Triosephosphate isomerase [Homo sapiens]	−1.32	0.02	Neurodegeneration [132]
	581	Q53G35	Phosphoglycerate mutase 1 (Brain) variant (Fragment) [Homo sapiens]	−1.6	0.043	AD [133]
Oxidative phosphorylation pathway	518	B3KP20	cDNA FLJ30970 fis, clone HEART2000444, highly similar to Homo sapiens phospholysine phosphohistidine inorganic pyrophosphate phosphatase (LHPP), mRNA [Homo sapiens]	1.57	0.043	AD [134]
	522	P36543	V-type proton ATPase subunit E 1 [Homo sapiens]	1.55	0.0068	
	658	O75947-2	(ATP5H)Isoform 2 of O75947. [Homo sapiens]	1.48	0.015	
	587	P47985	Cytochrome b-c1 complex subunit Rieske, mitochondrial[Homo sapiens]	1.38	0.013	
	634	O96000	NADH dehydrogenase (ubiquinone) 1 beta subcomplex subunit 10 [Homo sapiens]	−1.42	0.0059	
Nitrogen metabolism pathway	363	P15104	Glutamine synthetase[Homo sapiens]	1.85	0.0007	AD[135]
	578	P00918	Carbonic anhydrase 2 [Homo sapiens]	−3.11	0.009	Mental retardation, AD [136,137]
Arachidonic acid metabolism pathway	494	P16152	Carbonyl r Carbonyl reductase [NADPH] 1 [Homo sapiens]	2	0.014	AD [138]
Purine metabolism pathway	787	P22392	Nucleoside diphosphate kinase B [Homo sapiens]	−1.33	0.0061	DS, AD [139]
Arginine and proline metabolism pathway	369	P12532	Creatine kinase, ubiquitous mitochondrial [Homo sapiens]	1.47	0.0006	Alzheimer’s and Pick’s disease [140]
Glutathione metabolism pathway	624	P09211	Glutathione S-transferase P [Homo sapiens]	−1.64	0.024	Parkinson’s disease, AD [141,142]
MAPK signalling pathway	738	P16949	Stathmin [Homo sapiens]	1.48	0.031	DS and AD [143,144]
	608	P62993	Growth factor receptor-bound protein 2 [Homo sapiens]	1.29	0.04	AD [145]
Calcium signalling pathway	496	B4DKM5	cDNA FLJ60120, highly similar to Voltage-dependent anion-selective channel protein 2 [Homo sapiens]*	1.57	0.021	
	411	P50148	Guanine nucleotide-binding Protein G(o) subunit alpha [Homo sapiens]	1.36	0.026	Familial Alzheimer’s disease [146]
Axon guidance pathway	230	Q16555	Dihydropyrimidinase-related protein 2 [Homo sapiens]	1.57	0.025	AD[147]
Parkinson's disease pathway	384	Q7KYV2	H5 [Homo sapiens]*	1.37	0.035	Autosomal-recessive juvenile parkinsonism [148]
Antigen processing and presentation pathway	189	P11142	Heat shock cognate 71 kDa protein [Homo sapiens]	1.39	0.022	AD [149]
N/A	393	O00154	(ACOT7)Isoform 6 of O00154. [Homo sapiens]	1.64	0.0018	
	394	Q2TU84	Aspartate aminotransferase [Homo sapiens]	1.51	0.0048	
	350	P49411	Elongation factor Tu, mitochondrial [Homo sapiens]	1.35	0.008	Infantile Encephalopathy [150]
	723	P61601	Neurocalcin-delta [Homo sapiens]*	1.57	0.043	AD [151]
	475	B4DGP9	cDNA FLJ54102, highly similar to Beta-soluble NSF attachment protein [Homo sapiens]	1.53	0.033	
	458	P62879	Guanine nucleotide-binding protein G(I)/G(S)/G(T) subunit beta-2 [Homo sapiens]	1.73	0.0089	AD [152]
	784	A8MVL5	Putative uncharacterized protein PRDX5 [Homo sapiens]	−1.88	0.032	AD and parkinson [153,154]

Note: Most proteins in this table are involved in gene-ontology metabolic process except those proteins marked by *. Zhou et al., [1].

We have recently examined the genome wide mRNA and microRNA profiling using HIV+ patients with and without dementia. The findings indicated the significant involvement of axon guidance and its downstream signalling pathways (e.g. MAPK pathway) in HAD pathogenesis, which concurs with AD and PD as well [155]. Several specific miRNAs we found could differentiate between HAD and HIV non-dementia group, which interestingly also coincided with the miRNA seen previously in classical neurodegeneration [156-161]. The reason for the diversity of HAND is unknown, but it might due to host genetic factors. HIV might just play prompting role in the neurodegenerative process. This hypothesis needs to be elucidated by further studies to compare genetic differences between HAND patients with different neurodegenerative syndromes. However, there is a possibility that the viral, genetic and environmental factors might participate in as well. Nonetheless, taken together this is by far the most compelling evidence at the genomic, proteomic and gene regulation levels showing viral signatures in classical neurodegenerative diseases.

### e. Amyotrophic lateral sclerosis (ALS)

ALS is fatal neurodegenerative disease characterized by degeneration or loss of spinal cord and the cortical neurons. So far the cause for majority cases is unknown although genetic factors have been reported accounting for around 5% of all cases. Several viruses have been reported to be involved in ALS.

The involvement of retroviruses, including HIV, has been shown based on findings, which reported that ALS-like syndrome to occur in HIV and human T-cell leukemia virus type-1 (HTLV-1) infection [129,162] and MacGowan has shown that with antiretroviral therapy, an ALS-like disorder in HIV-positive patients can abate [163]. Infection with several other retroviruses infection has also been documented to cause ALS-like syndromes [164-167]. In addition, the reverse transcriptase (RT) has been found to be positive more frequently in ALS patients’ sera compared to that of control and the levels of the activity in ALS patients were comparable to that in HIV infected patients [168-170]. Intriguingly, RT activity is hardly detected in CSF of ALS patients and XMRV sequence is not detectable in blood [170]. This result is consistent with another work [169]. RT is an essential retroviral enzyme product that allows viral genome to integrate into the host genome and reproduce its progeny as an active virus upon activation signal. Therefore, its frequent seropositivity and high activity in patients with ALS might signal the systemic retroviral replication. The reason for its absence in CSF is unknown. The plausible explanation would be RT is released from lysed cell and CSF is a cell-free compartment. Recently, expression of distinctive HERV-K pol-like sequences has been demonstrated preferentially in prefrontal and sensory cortex, areas adjacent to the motor cortex [171]. Intriguingly, they could only identify the RT in neurons rather than other cell types.

## Possible mechanisms in viral pathogenesis that explain neurodegenerative disorders

### Viral neurotropism

It is well known that many viruses can enter the CNS, but the mechanism is not well defined. There are two distinct routes: one is hematogenous dissemination and the other is neuronal retrograde dissemination. The first one is better accepted that virus enter the brain by crossing blood brain barrier via several pathways: transcytosis; transition by infected endothelial cells, passage through the blood–cerebral spinal fluid (CSF) barrier of the choroid plexus, and Trojan horse (utilizing infected microphage and microglia). This hypothesis is well documented in HAD and it has been reported in other viral neurodegenerative disease as well [172]. However, there is more evidence for the latter pathway recently. It has been shown that influenza virus (A/WSN/33 strain) can enter the CNS via olfactory epithelium [173], and possibly cranial nerves as well including the vagus and trigeminal nerves [174,175]. Recently, Harberts et al. has shown HHV-6 entry into CNS through that pathway as well [176]. HSV and rabies virus have also been documented to take this route into CNS [177]. Other study has shown that A/Vietnam/1203/04 H5N1 virus can enter CNS from peripheral nervous system [110]. We also found in HAD patients, mid part of brain as well as spinal cords were heavily involved in HIV productive infection [124]. Our findings are consistent with their findings and we have hypothesized that HIV might transmit into the brain from the peripheral nervous system (PNS).

### Viruses, inflammation, immune activation and neurodegeneration

In modern times, we have seen a dramatic increase in mean life span, which is paralleled by an epidemic of chronic disease usually associated with ageing [178]. In spite of the immune privileged status of the CNS, it is still well known that dynamic immune and inflammatory responses result from a variety of insults in this compartment, and viral infection accounts for one of them [179]. Inflammatory responses also appear to be the prevalent triggering mechanism driving tissue damage associated with different age-related diseases. Age-dependent up-regulation of the inflammatory response is primarily a consequence of chronic antigenic stress, which in turn bombards the innate immune system throughout lifetime of an individual, and possibly triggering the onset of inflammatory disease. A considerable body of experimental and clinical evidence suggests that the immune system plays a definitive role, at variable degrees, in almost all age-related or associated diseases [180].

It is well known that viruses can prime the immune system to respond aberrantly. Immune and inflammatory response is a double-edged sword in neurodegenerative disease depending on the duration of the inflammatory response. Usually, acute neuro-inflammatory response includes an immediate and short-lived activation of the innate immune system within the CNS [181,182]. It is beneficial to the CNS, as it minimizes injury, repairs and cleans the damaged tissue. In contrast, chronic inflammation is characterized by long-standing activation of microglia and sustained release of inflammatory mediators, which lead to increase oxidative and nitrosative stress, subsequently perpetuating inflammatory cycle [183]. Moreover, chronic neuro-inflammation also contributes to further prolonged inflammation [179,184]. Therefore, chronic neuro-inflammation is highly detrimental to long term neuronal survival compared to major protective role of acute neuro-inflammation. Several neurodegenerative diseases have been reported to be associated with chronic neuro-inflammation, including AD, PD, MS, ALS, etc. [185-188]. Furthermore, the dysregulated systemic immune responses have been shown to serve as a signature for early detection of AD [189] and long-term use of non-steroidal anti-inflammatory drugs (NSAIDs), in particular ibuprofen, have been proven to be effective preventing the development of AD [190]. As a key feature of chronic neuro-inflammation, microglia activation certainly plays a central role in the pathophysiology of neurodegenerative diseases. It has been reported that IL-1 positive activated microglia are co-localized with both αβ plaques and neurofibrillary tangles in AD brains and lead to excessive tau phosphorylation [188]. The activated microglia also present in the areas where degenerating motor neurons are present in both ALS patients and animal models [191,192]. In addition, positron emission tomography (PET) studies have confirmed the correlation between the increased activated microglia in the motor cortex and upper motor neuron symptoms [193]. Significantly elevated microglia activation in the pons, basal ganglia, striatum, and frontal and temporal cortical regions of PD brains has also been shown by PET study [194]. It has been shown that several neurotropic viruses, such as HIV and HSV, can trigger long-term neuro-immune activation [195], which might be one of the underlying mechanisms of viral neurodegenerative diseases.

Neuroinflammation can be either a cause, or a consequence of chronic oxidative stress. Therefore, in this context it is important to mention that oxidative stress is one of the main features of neurodegenerative diseases and it binds all neurological and neurodegenerative diseases together. The oxidative stress causes lipid, protein, and genetic structural alterations that result in degeneration of nerves. Although the oxidative stress has variety of causes, such as environmental toxic insults in combination with genetic factors, it is also attributed to the involvement of pathogenic etiologies that cause neurodegenerative diseases. Chronic viral and pathogen-mediated proteins in the presence of excess reactive oxygen species can induce pathologic changes in neural tissue and lead to chronic inflammation of the brain, as seen in classical neurodegenerative diseases.

### Viruses, innate immunity/adaptive immune system and neurodegeneration

Innate immunity is an evolutionarily ancient system that provides organisms with immediately available defense mechanisms through recognition of pathogen-associated molecular patterns. Evidence shows that the dysregulation of innate immune system has been seen broadly in classical neurodegenerative diseases, such as AD, PD and Schizophrenia and it is an excellent indication of pathogen-related footprints on neurodegeneration. Lenhardt et al., have shown that in the CNS, specific activation of innate immunity through a Toll-like receptor 4 (TLR4)-dependent pathway leads to neurodegeneration [196]. It is important to iterate that innate immunity is a constitutive component of the central nervous system (CNS) and heavily depends on resident myeloid cells, the microglia. Recently emerging evidence suggests that the most abundant glial cell population of the CNS, the astrocyte, participates in the local innate immune response triggered as a consequence of constant insults they go through during infection or inflammation and it is important to state that they have been shown to be reservoirs of HIV-1 and play a significant role in virus-mediated neurodegeneration. Interestingly, astrocytes display an array of receptors, which are involved in innate immunity, including Toll-like receptors, nucleotide-binding oligomerization domains, etc.

Fassbender et al., have shown that the detection of CD14 overexpression in brains of APP transgenic mice points towards the key role of innate immunity receptor CD14 in significantly contributing to the neuroinflammatory responses in AD. CD14 recognizes the pattern associated with the ß-sheet fibrillar conformation of Aß, as this receptor interacts with fibrillar but not with non-fibrillar Aß. The affinity of fibrillar Aß to CD14 is ~50-fold lower than that to LPS. However, since in contrast to LPS the AD brains contain very high concentrations of Aß fibrils (in contrast to LPS) for years and decades, it is likely that even at this submaximal affinity, this interaction is sufficient to maintain a chronic neuro-inflammation. Overall, this study provides the basis for possible structural mimicry between highly hydrophobic fibrillar Aß and pathogen-associated molecular patterns contributing to neuro-inflammation in AD and opens the therapeutically relevant perspective that the enormous progress being made in the field of innate immunity could be extended to AD and other neurodegenerative disorder research [197]. Previous studies have suggested that pathogens could be involved in AD too. CD33, one of four genes found to be significantly associated with AD in a recent genome-wide association screening (Bertram et al., 2008), is involved in activating the innate immune system. But perhaps the most prominent example of a dementia associated with viral infection is HIV-associated dementia, which shows the innate immune system involvement and proteomic overlaps with AD, PD and Schizophrenia (Table 2) [1].

### Targeting immune system: a new way to treat neurodegeneration

In the context of innate immune system involvement in classical neurodegeneration, it is important to mention that scientists in France and the US discovered that a type of immune system cell may facilitate the development of PD and it has been suggested that targeting part of the immune system with drugs could be a new way to treat the disease [198]. In their study, Brochard and colleagues used postmortem evidence of human patients to show that CD8+ and CD4+ T cells, but not B cells, had invaded their brains. They then used mice that had been given a neurotoxin to induce symptoms of Parkinson’s (the MPTP mouse model) to show that it was almost exclusively the CD4+ T cells that arbitrated the accelerated death of dopamine cells. This only happened when the FasL cell death triggering protein was expressed, and not when the IFN-γ inflammatory cytokine was expressed. This study suggests both activated microglia and T lymphocytes do make significant contribution to neurodegeneration in PD.

Elevated T cell responses to specific CNS antigen, or shift in CD4+ and CD8+ T cell population in the periphery as well as CNS has also been observed in neurodegenerative disorders [199-201]. Both CD4 and CD8+ T cells can be detrimental and protective to the CNS. However, in that T cells do not significantly accumulate in the brain undergoing neurodegeneration and more importantly T cells directed to myelin or neuronal antigens can also be found in healthy control subjects [202,203], therefore it is difficult to conclude any direct involvement of T cells as a causative factor in neurodegenerative diseases. Nevertheless, the alteration of CD4+ and CD8+ T cells observed in the periphery in neurodegenerative diseases indicates that there is persistent antigenic challenge and T cells are playing a role in neurodegenerative diseases. It is worthy to note that the ratio of CD8+ to CD4+ T cell or the shift to a Tc1/Th1-type immune response may contribute to the harmful brain inflammatory reaction.

Further the evidence solidifying the role of immune system in NDs, is the presence of antibodies against neuronal antigen observed in neurodegenerative diseases and some of them are pathogenic [2]. The mechanisms of how these antibodies arise remain unknown. One plausible explanation is the possible homology between some pathogen antigens and human neuronal antigens.

Overall, together these studies give further evidence that the immune system can both protect and attack the brain. Whether this is a generalized possibility across all neurodegenerative disorders is an open area of investigation. Although this study, combined with several other studies discussed in this review, cumulatively implicate pathogen and innate immune system involvement in classical neurodegeneration, which upon further studies will open the door to developing new treatments that target the immune system for treating classical neurodegenerative disorders, especially if the NDs occur at younger age because treating the ageing immune system may not yield the same effect.

### Further recent evidence showing the role of immune system in schizophrenia

In case of schizophrenia, both the unspecific and the specific arms of the immune system seem to be involved in the dysfunction of the immune system in schizophrenia. The unspecific, “innate” immune system shows signs of over-activation in un-medicated schizophrenic patients, as indicated by increased monocytes and γδ-cells. Increased levels of interleukin-6 (IL-6) and the activation of the IL-6 system in schizophrenia might be the result of the activation of monocytes/macrophages, too. On the other hand, several parameters of the specific cellular immune system are blunted, such as, for example, the decreased T helper-1 (TH-1)-related immune parameters in schizophrenic patients both *in vitro* and *in vivo*. It seems that a TH-1/TH-2 imbalance with a shift to the TH-2 system is associated with schizophrenia [204]. Three schizophrenia genetics research consortia, each funded in part by NIMH, have reported separately on their genome-wide association studies (http://www.genome.gov/20019523) online July 1, 2009, in the journal Nature. However, the SGENE, International Schizophrenia (ISC) and Molecular Genetics of Schizophrenia (MGS) consortia shared their results-making possible meta-analyses of a combined sample totaling 8,014 cases and 19,090 controls. Interestingly, all three studies implicate an area of Chromosome 6 (6p22.1), which is known to harbor genes involved in immunity and controlling how and when genes turn on and off. This hotspot of association might help to explain how environmental factors and infection by pathogens affect risk for schizophrenia. Recently, genetic data from multiple large cohorts of patients were published in ‘Nature’ showing that different gene loci located on chromosome 6p22.1 are the most probable susceptibility genes for schizophrenia [3]. This region includes several genes of interest, which are related to the immune function. The strongest evidence for association was observed in or near a cluster of histone protein genes which could be relevant through their roles in regulation of DNA transcription or repair, i.e. in epigenetics [205], or their direct role in antimicrobial defense [206]. Moreover, several genes of the HLA complex, which regulate the immune function and already earlier have been discussed to be involved in schizophrenia, are located in these regions [207].

Although an immune dysfunction and the involvement of infectious agents in the pathophysiology of schizophrenia are discussed since decades, the field never came into the mainstream of research. These genetic findings and further recent interesting observations, however, may contribute shifting research into the direction of immunological alterations and inflammation as cause for schizophrenia. Although the involvement of infectious agents has long been suspected in schizophrenia, results from animal models indicate that the immune response determines the risk for schizophrenia [208].

It is well known that the molecules participating in both innate and adaptive immune responses have been shown to be induced in a wide diversity of neurological disorders, which include AD, MS, HD, PD, and ALS all discussed in this review. The recent evidence on immune response within the brain actually points towards host immune mechanisms could be the actual culprit or in other word etiologically linked to the manifestation of neurodegenerative diseases. Further, this also suggests that the primary causes of a large majority of neurologic disorders seen in humans could originate outside the central nervous system. Some infectious agents are known to gain entry into the CNS within infected migratory macrophages, which traverse the blood brain barrier by transcytosis or by intra-neuronal transfer from peripheral nerves. In case of various viruses, especially the ones associated with NDD, the systemic nature of these pathogens is able to trigger immune responses, along with affecting the immune system and other organ systems including the brain, resulting in neurologic manifestation.

Although the underlying mechanisms of HAD are still uncertain, the cumulative evidence has pointed out that HIV might cause immune-senescence. First of all, it is evident that the age of HIV+ patients who manifest dementia and Alzheimer’s disease, which is 15 years (50–52 years) less than HIV- individuals who manifest AD (Average age 65–85). Second, apart from dementia, HIV can also lead to other aged diseases, which is closely related to immune-senescence, such as cardiovascular diseases and cancer, as HIV interferes with metabolic and oxidative phosphorylation pathways [209]. Third, it has been shown that HIV infection can induce T lymphocytes immune-senescence, which is indicated by deteriorated shortening of telomeres in CD8+ CD28- T cells while no significant change in CD4+ T cells [210,211] and this is highly similar with what has been seen in the elderly. In addition, a marker of immunosenescence, CD57, can also be detected during initial stage of HIV infection [212]. Furthermore, HIV infection can induce similar alterations to innate immune cells, including monocytes, NK cells, dendritic cells and neutrophils, compared to that induced by ageing [213]. In this context it is important to mention that even though HIV shares anatomical, clinical and proteomic overlaps with both AD and PD, the involvement of virus or any pathogen in classical neurodegeneration has not been proven.

### Dysregulated metabolic and transport machinery in NDs: evidence for viral interference

It is well known that metabolic pathways have an integral involvement in neurodegenerative process in humans. With ageing, there is up regulation of both oxidative stress and metabolic pathways in the human brain and more so during the manifestation of neurological disease. The significant dysregulation of both these pathways is apparent in a variety of viruses, which include, HIV, HCV, Influenza, etc. It has been shown that the impairment of energy metabolism can exacerbate synaptic dysfunction, neuronal injury, which together may lead to neurodegenerative disorders [214]. Viruses are completely reliant on their host’s cellular nucleic acids, proteins and membrane, as well as energy to enable viral synthesis. Disturbance/dysregulation of several core metabolic pathways have been reported by viral infections, including glycolysis and lipid metabolism, which all play a very important role in neurodegenerative diseases [215]. Viruses use several means to interfere with host metabolism, including manipulating host gene expression; inhibiting host cellular process (such as RNA and protein synthesis); impairing mitochondrial function and interfering ATP production [215]. Indirect evidence also comes neuro-proteomic analysis of HIV+ patients with and without dementia, which showed the first evidence that >90% of the protein candidates identified in the HAD brains, particularly in the oxidative phosphorylation (OXPHOS) and glycolysis pathways appear also to over represent in non-viral mediated neurodegenerative diseases, such as vascular dementia, AD, PD and amyotrophic lateral sclerosis (ALS) [1], a study which is highly significant as it shows the ultimate footprints of a virus on neurodegenerative disease and its overlap with classical forms of neurodegeneration has considerable potential for treatments for NDs in humans.

Viral infection also interferes with host transport machinery, whose alterations are cardinal feature of neurodegenerative diseases, eg. neurofibrillary changes in AD, Lewy bodies in PD, and different types of filamentous inclusion bodies in motor neuron disease. It has been well documented that viral proteins can modulate the structure and function of the actin cytoskeleton to initiate, sustain and spread infections [216].

## Conclusions

Based on all the evidence and controversies discussed in this review, one single fact emerges out suggesting that viruses can be causative agents or at least co-factors of some of neurodegenerative diseases, if not all. Even though the virus involvement in human NDs is hotly debated and disputed, the take home message from the studies complied in this review is the mechanistic similarities between viral and non-viral neurodegenerative diseases, which further imply that viruses can act as excellent models in understanding neurodegeneration in humans. A critical understanding of these overlaps between viral and non-viral NDs may lead to new strategies to treat human NDs. But there is still a lot of work to be done before pathogen link to classical neurodegenerative diseases becomes fully concrete.

## Competing interest

All authors declare that they have no competing interests.

## Authors’ contributions

NKS suggested and supervised the idea. JZ and NKS worked on the outline of the review in consultation with MMS. JZ executed the idea, wrote together with NKS and MMS. MMS offered considerable help with the sections on Herpes virus families and their involvement in neurodegeneration. NKS critically reviewed, corrected and guided the completion of the review. All authors read and approved the final manuscript.
